# Enhanced Early Detection and Precision Monitoring of Rubber Tree Powdery Mildew Pathogen *Erysiphe quercicola* Using Quantitative PCR and Droplet Digital PCR

**DOI:** 10.3390/jof12030185

**Published:** 2026-03-05

**Authors:** Xiaoyu Liang, Deyu Feng, Mengyuan Xiong, Shaoyao Zhou, Lifeng Wang, Shanying Zhang, Meng Wang, Yu Zhang

**Affiliations:** 1 State Key Laboratory of Tropical Crop Breeding, Sanya Institute of Breeding and Multiplication, School of Tropical Agriculture and Forestry, Hainan University, Sanya 572025, China; liang2017@hainanu.edu.cn (X.L.);; 2 Key Laboratory of Biology and Genetic Resources of Rubber Tree, Ministry of Agriculture and Rural Affairs, State Key Laboratory Incubation Base for Cultivation & Physiology of Tropical Crops, Rubber Research Institute, Chinese Academy of Tropical Agricultural Sciences, Haikou 571101, China

**Keywords:** rubber tree powdery mildew, *Erysiphe quercicola*, qPCR, ddPCR, PMA treatment, precision monitoring

## Abstract

Rubber trees are crucial to the global industrial economy, but they are facing the threat of powdery mildew caused by *Erysiphe quercicola*. Effective management of this disease depends on early detection. However, traditional monitoring methods are labor-intensive and often inaccurate. This limitation underscores the need for more precise and efficient techniques. This study developed and validated an integrated molecular detection platform that combines quantitative PCR (qPCR), droplet digital PCR (ddPCR), and propidium monoazide (PMA) treatments. The platform demonstrated a robust detection range, accurately quantifying *E. quercicola* at concentrations as low as 10 spores/mL spore DNA and 10^−5^ ng/μL mycelial DNA. Additionally, the system distinguished viable from non-viable spores and detected *E. quercicola* mycelia in both asymptomatic leaves and aged lesions, significantly enhancing early-stage detection and disease monitoring. This technology also helps assess the efficacy of fungicides against powdery mildew, potentially reducing the use of chemicals and their environmental impact. By improving early diagnosis and disease management, this approach promises to reduce dependence on fungicides and mitigate economic and environmental impacts, highlighting the enormous potential of advanced molecular technologies in sustainable agricultural practices in rubber plantations.

## 1. Introduction

Rubber trees (*Hevea brasilensis*) are vital to the global industrial economy. They thrive in the warm and humid environment of tropical regions and are the main source of latex in the area. However, these ideal growing conditions also promote the proliferation of *Erysiphe quercicola*, the pathogen responsible for powdery mildew [1]. This disease poses a significant threat, especially in Asia’s vast rubber plantations, leading to considerable reductions in both yield and latex quality [2]. The economic implications, coupled with environmental concerns such as soil and water contamination due to excessive fungicide use, underscore the urgent need for precise and sustainable disease management strategies in rubber plantations [3].

Powdery mildew in rubber trees is strongly influenced by factors such as meteorological conditions, plant developmental stages, and the presence of the pathogen [4]. Given the economic importance of rubber plantations, especially in tropical regions, effective disease management is critical. Early detection is essential for timely interventions, allowing for disease control before visible symptoms appear, thereby curbing pathogen spread and minimizing yield losses. However, current monitoring methods—such as visual inspections, field surveys, microscopic analyses, and pathogen isolation—are labor-intensive and prone to human error, leading to inconsistent results. These traditional approaches often lack the precision required for managing large-scale plantations [5]. While spore trapping offers some improvements for monitoring *E. quercicola* from rubber plantations [6], environmental factors like dust and particulates can compromise spore count accuracy, limiting its effectiveness [7]. Moreover, although PCR-based molecular detection is used for diagnosing rubber tree powdery mildew [8], its low sensitivity restricts its application for early detection of mycelia and spores. This highlights the urgent need for more advanced and reliable monitoring technologies capable of overcoming these limitations, providing timely and accurate data to optimize disease management strategies.

To address these challenges, quantitative PCR (qPCR) offers sensitive, specific, and quantitative pathogen detection [9]. The introduction of qPCR has revolutionized pathogen detection by enabling real-time monitoring of DNA amplification and allowing for the detection of pathogen DNA even before visible symptoms appear [10]. This approach facilitates early intervention and reduces fungicide reliance. Moreover, the ability to quantify the pathogen load helps assess the severity of the infection and tailor control measures accordingly. QPCR is currently regarded as a reliable and widely used technique for the early detection of diseases. Although the use of standard curves and melt curve analyses is essential during the assay validation phase, once a marker gene has been properly validated, the specificity and reliability of the amplified product can be ensured [11]. In this context, rather than questioning the robustness of qPCR, it is more appropriate to acknowledge that complementary molecular approaches exist, such as droplet digital PCR (ddPCR), which has also demonstrated high efficiency and accuracy for disease detection [12,13]. However, research on the use of qPCR and ddPCR for detecting *E. quercicola* and their potential applications in controlling rubber tree powdery mildew is lacking and requires further evaluation.

Propidium monoazide (PMA) is a highly affine photosensitive dye that, under light treatment conditions, covalently cross-links with the DNA of dead cells to form robust carbon-nitrogen bonds, resulting in precipitates that block DNA amplification in PCR. PMA does not penetrate cell membranes, thereby selectively modifying the DNA of dead cells with damaged membranes [14]. Any unbound PMA in the solution reacts with water molecules under intense light to decompose into hydroxylamine compounds with no cross-linking activity, ensuring that it does not affect PCR amplification. Combining PMA with qPCR and ddPCR (PMA-qPCR and PMA-ddPCR) techniques enables the detection of a range of sample types, including bacteria, yeasts, fungi, viruses, and eukaryotic cells [11,15,16]. It is urgent to evaluate and apply this combined technology’s capability to detect viable mycelia and spores of *E. quercicola*.

Therefore, the objectives of this study were to: (i) design qPCR and ddPCR protocols for detecting and quantifying *E. quercicola* in spore traps and rubber leaves, (ii) compare the accuracy, sensitivity, and stability of ddPCR with qPCR in detecting mycelia and spores of *E. quercicola*, and (iii) evaluate the effectiveness of PMA-qPCR and PMA-ddPCR in identifying reinfection sources and measuring fungicidal control efficacy against powdery mildew. By optimizing qPCR and ddPCR protocols and integrating PMA treatments, this study aims to enhance detection and precise disease management, improving powdery mildew control while reducing labor and economic costs.

## 2. Materials and Methods

### 2.1. Plant Material and Fungal Strains

The ‘Reyan 73397’ bud-grafted saplings, aged ten months, were sourced from the superior seedling breeding base of the Rubber Research Institute, Chinese Academy of Tropical Agricultural Sciences. These saplings were planted in 3-gallon pots filled with peat soil and fertilized biweekly with Hoagland’s solution. Once the saplings had developed two clusters of leaves, reached a uniform height, demonstrated good growth vigor, and exhibited bronze-colored leaves, they were selected for the experiments. All fungal strains used in this study were obtained through monospore isolation in our laboratory and were molecularly identified (Table S1).

### 2.2. E. quercicola Culture and Spore Suspension Preparation

The bud-grafted saplings were cultivated at 28 °C until they developed 2 to 3 cm long bronze leaves, then transferred to a powdery mildew inoculation chamber maintained at 18–20 °C with 70–90% relative humidity, under a 16-h light/8-h dark cycle. After 12 to 16 days, *E. quercicola* spores were harvested from the leaves. The spores were gently shaken off the leaves 24 h before use and collected with a clean, soft brush. They were then suspended in 1 mL sterilized deionized water and purified by centrifugation. A ten-fold serial dilution was performed using a hemocytometer to prepare spore suspensions with concentrations ranging from 10 to 10^5^ spores/mL.

### 2.3. DNA Extraction

For mycelial DNA extraction, *E. quercicola* was collected directly from fresh lesions 8–10 days post-inoculation using a sterilized soft brush to minimize contamination from plant material. For spore DNA extraction, *E. quercicola* was gathered directly from older lesions 12–16 days post-inoculation and from spore traps. Both types of samples were then suspended in sterilized deionized water and purified by centrifugation, ground with liquid nitrogen to break down cell walls and release DNA, following the established protocol in our laboratory [17]. Genomic DNA (gDNA) was extracted from *E. quercicola* mycelia and rubber tree leaves using the E.Z.N.A.^®^ HP Fungal DNA Kit and the E.Z.N.A.^®^ HP Plant DNA Kit (Omega, Knoxville, TN, USA), following the manufacturer’s instructions.

### 2.4. Design and Screening of Specific Primers

Primers ranging from 15 to 25 nucleotides were designed based on the specific conserved sequence of *E. quercicola* (Genbank: KP171513.1) using Primer Premier 5 software. Three pairs of specific quantitative primers were selected (Table S2). The specificity of these primers was verified through PCR, qPCR, and ddPCR with six different fungi and rubber trees. The template DNA was diluted to 50 ng/μL for the PCR reaction, which included 1 μL of genomic DNA, 10 μL of 2 × Magic Green Taq Mix (Vazyme, Nanjing, China), 0.5 μL of each primer, and 8 μL of deionized water, totaling 20 μL. The PCR protocol featured an initial denaturation at 95 °C for 5 min, followed by 35 cycles at 95 °C for 45 s, 56 °C for 30 s, and 72 °C for 45 s, with a final extension at 72 °C for 7 min. The optimal annealing temperature for the PCR assay was assessed using a thermal gradient between 50 and 65 °C for a 30-s extension time.

### 2.5. Cloning of Target Sequences

The expected fragments of *E. quercicola* amplified by the specific primers were gel-purified using a Gel Extraction Kit (Omega, USA) and ligated into a TOPO vector. After cloning in *E. coli DH5α* competent cells, plasmid DNA (pDNA) was extracted using a Plasmid Mini Kit (Omega, USA). The quality and concentration of the pDNA were confirmed by sequencing and measured using a Nanodrop spectrophotometer (Implen, Munich, Germany). The copy concentration was calculated with the formula: Number of copies = (amount × 6.022 × 10^23^)/(length × 10^9^ × 650).

### 2.6. qPCR Parameters

The qPCR reaction mixtures contained 1 μL of template gDNA, 10 μL of qPCR SYBR Green Master Mix (Vazyme, China), 0.5 μL of each primer (10 μM), and an appropriate volume of deionized water to reach a total reaction volume of 20 μL. Amplification was performed using a Bio-Rad CFX96 (Bio-Rad, Hercules, CA, USA) with the following thermal cycling parameters: initial denaturation at 95 °C for 30 s, followed by 40 cycles of 95 °C for 5 s, and annealing at 56 °C for 35 s. Fluorescence was measured at the end of each extension step. The optimal annealing temperature for the qPCR assay was assessed using a thermal gradient ranging from 50 to 65 °C for a 35-s extension time.

### 2.7. ddPCR Parameters

The ddPCR experiments were conducted using the QX200 ddPCR System (Bio-Rad, Hercules, CA, USA). The reaction mixture consisted of 12.5 μL of EvaGreen Supermix (Bio-Rad), 1.25 μL of each primer (10 μM), 1 μL of template gDNA, and an appropriate volume of double-distilled water (ddH_2_O) to achieve a final volume of 20 μL. A non-template control (NTC) was included in each experiment using 1 μL of ddH_2_O instead of the DNA template. The mixture was loaded into a disposable plastic cartridge with 70 μL of droplet generation oil (Bio-Rad) and processed in the droplet generator (Bio-Rad). Following droplet generation, the samples were transferred to a 96-well PCR plate and amplified on a Veriti 96-well thermal cycler with the following protocol: initial denaturation at 95 °C for 5 min, 40 cycles of denaturation at 95 °C for 30 s, and annealing at 56 °C for 60 s, followed by a final incubation at 90 °C for 5 min. The droplets were then read in the Droplet Reader (Bio-Rad), and the data were analyzed with QuantaSoft software v2.2 (Bio-Rad, Hercules, CA, USA). The absolute concentration of each sample was determined by calculating the ratio of positive to total droplets and applying Poisson statistics. The optimal annealing temperature was assessed using a thermal gradient between 50 and 65 °C for a 60-s extension time.

### 2.8. Linearity, Dynamic Range, Stability and Tolerance Tests

The linearity, dynamic range, and stability of the assays were evaluated using a 10-fold dilution series of DNA for a plasmid containing the target fragment (10^−2^ to 10^8^ copies/μL), *E. quercicola* mycelia (5 × 10^−5^ to 50 ng/μL), and spores (10^1^ to 10^5^ spores /mL). These dilutions were tested in triplicate within a single run and across three independent runs. The results were utilized to establish linear regression curves between the logarithm of the template concentration and the quantification values, and the coefficient of variation (CV) was calculated from the standard deviation and mean of the triplicate measurements. Tolerance experiments involved diluting isopropanol into six gradients (0, 3, 6, 9, 10, 15, and 20 times) and comparing the results with the no-template control (NTC). Similarly, rubber tree sap was diluted to the same gradients and assessed alongside the NTC. These dilutions were used to replace ddH_2_O in the reaction systems for subsequent qPCR and ddPCR experiments.

### 2.9. Quantification of E. quercicola Spores in Spore Trap Samples

During the epidemic period of rubber tree powdery mildew (from February 2023 to April 2023), eight spore samples were collected from Burkard spore traps placed in forest sections located in Baoting, Hainan (18°23′00″ N, 109°48′00″ E) and Jinghong, Yunnan (22°01′00″ N, 100°49′00″ E), with samples taken every 3 days. After being transported at low temperatures, the spores were stored in a freezer at −80 °C. DNA of *E. quercicola* spore was extracted from these samples and quantified using qPCR and ddPCR. Subsequently, the test results were correlated with observations made under a microscope. It was assumed that all spores contained within 0.99 m^3^ of air were captured over a one-hour interval. The airborne spore concentration over a three-day period (C) was estimated (spores/m^3^) using the following formula:

$$C\left(spores/m³\right)=\frac{N_{spores}\times \frac{A_{slide}}{A_{view}}}{Q_{air}\times T_{sampling}}$$

where: *N_spores_* is the number of spores counted under the microscope, *A_view_* is the area of the microscope’s field of view, *A_slide_* is the total effective sampling area on the slide, *Q_air_* is the air flow rate (in cubic meters per hour), *T_sampling_* is the total sampling time.

### 2.10. Quantification of E. quercicola in Rubber Tree Leaves

To simulate natural infection conditions, five rubber seedlings at the bronze stage were placed among heavily infected plants producing high quantities of *E. quercicola* spores. To estimate the spore concentration within the chamber, petroleum jelly-coated slides were positioned near the plants to capture airborne spores. These slides were examined microscopically, enabling calculation of the ambient spore concentration. The seedlings were maintained in an enclosed growth chamber for 8 days, with daily monitoring for symptom development. Pathogen presence was confirmed by detecting *E. quercicola* on three selected leaves. Genomic DNA was extracted following the previously described methods and analyzed using qPCR and ddPCR to determine the earliest point of *E. quercicola* DNA detection. Visual assessments of symptoms were also conducted to compare the appearance of visible signs with the detection limits of the molecular assays.

### 2.11. Optimization of PMA Treatment

The PMA treatment system for detecting *E. quercicola* was optimized by exploring three inactivation methods: ultra-low temperature treatment, where 50 mg of *E. quercicola* conidia were suspended in 2 mL of deionized water at a concentration of 5 × 10^5^ spores/mL and frozen at −180 °C for 24 h; boiling water bath treatment, where the spore suspension was heated at 95 °C for 10 min; and isopropanol treatment, where the spores were treated with 70% isopropanol by volume for 30 min and then centrifuged at 12,000 rpm for 10 min to discard the supernatant and resuspend the spores. Different concentrations of PMA (0, 10, 20, 40, 60, 80 µg/mL) were tested on live and dead samples to determine the optimal concentration that could effectively inhibit the amplification of live fungi in qPCR. Additionally, the best inactivation method and PMA concentration were used to expose dead samples to light using a 400 W halogen lamp for 0 to 25 min to select the optimal light reaction time through qPCR. The sensitivity of PMA-qPCR and PMA-ddPCR was compared by diluting the live spore suspension into six gradients (10–10^5^ spores/mL) for PMA treatment. Mixtures of live and dead spores at a concentration of 5 × 10^5^ spores/mL were prepared in ratios of 0, 25, 50, 75, and 100%, with a total volume of 200 μL, treated according to the optimized PMA system, and tested with qPCR and ddPCR, maintaining a consistent reaction system and protocol.

### 2.12. Quantification of Viable E. quercicola in Aged Lesions of Rubber Tree Powdery Mildew

Fresh and aged powdery mildew spots from rubber tree leaves artificially inoculated indoors were collected (Figure S1). The diseased tissue from each sample was cut into 1 × 1 mm pieces. After PMA treatment, and without treatment, samples were amplified using qPCR and ddPCR to quantify the results of both methods.

### 2.13. Quantification of Viable E. quercicola in Fungicide Treated Leaves of Rubber Tree

Rubber seedlings at the bronze stage were inoculated with *E. quercicola* at a concentration of 10^5^ spores/mL. Once the plants exhibited symptoms, the diseased leaves were sprayed with a 15% triadimefon wettable powder. Samples of the powdery mildew spots were collected at specific intervals (0, 5, 10, 15, 30, 60, 120, 180, 240, and 300 min) over ten treatment periods, with three replicates each. The samples were ground into powder using liquid nitrogen, and 50 mg of the powder was quickly weighed into a 2 mL sterile centrifuge tube. One tube was used directly for DNA extraction, while another tube had 1 mL of sterile ddH_2_O added and was then shaken to mix. The tubes were then wrapped in aluminum foil to protect them from light, and DNA was extracted after PMA treatment. Samples not treated with fungicide served as controls for PMA-qPCR and PMA-ddPCR analysis.

### 2.14. Statistical Analysis

Statistical analyses were performed using SPSS software (version 22, IBM). Linear regression was utilized to assess the linearity of qPCR and ddPCR data, with coefficients of determination (R^2^) calculated using Origin 2018 software. The least significant difference (LSD) test was applied for multiple comparisons among treatments, identifying significant differences at *p* < 0.05. Independent *t*-tests were also conducted to compare means between two distinct groups for targeted analyses. Coefficients of variation (CV) were calculated to evaluate the reproducibility and stability of the assays across various runs.

## 3. Results

### 3.1. Development and Validation of DQ-25 Primers for Specific Detection of E. quercicola Using qPCR and ddPCR

**Figure 1:**
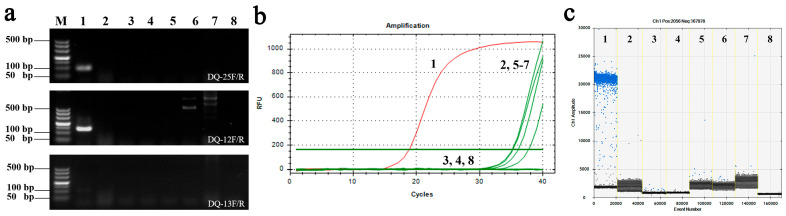
Specificity validation of primer DQ-25 by PCR (**a**), qPCR (**b**), and ddPCR (**c**). M: DL500 DNA Marker; 1. *E. quercicola*; 2. Rubber tree; 3. *E. polygoni*; 4. *Corynespora cassiicola*; 5. *Podosphaera xanthii*; 6. *Colletotrichum siamense*; 7. *Phytophthora palmivora*; 8. nontemplate control (NTC).

Specific conservative sequences of *E. quercicola* (Genbank: KP171513.1) were targeted for the design of three pairs of primers for qPCR and ddPCR detection. These primers were initially used to amplify DNA from *E. quercicola* and were compared with DNA from rubber trees, pathogens causing powdery mildew in two other crops, and three common pathogens of rubber trees to verify the primers’ specificity. As shown in (Figure 1a), the DQ-25 primers exhibited good specificity for *E. quercicola*, while no specific fragments were produced with other target DNA templates. Subsequently, the annealing temperature for the DQ-25 primers was optimized, with temperatures ranging from 52 °C to 55.7 °C yielding good amplification results in PCR, qPCR, and ddPCR processes (Figure S2; Table S3). The optimal annealing temperature was determined to be 55.7 °C. Specificity testing of qPCR and ddPCR was conducted using 50 ng/μL of *E. quercicola* DNA and non-target DNA. In the 40 amplification cycles of qPCR, the Cq value of *E. quercicola* was 18.79, while no Cq values were detected for the other non-target templates and blank controls. (Figure 1b). In the ddPCR results, the amplification value for *E. quercicola* was 236 copies/μL, with no amplification signal detected in other non-target templates (Figure 1c). These results indicate that the DQ-25 primers are highly specific and can distinguish between *E. quercicola*, rubber tree, and other related pathogens. Moreover, the amplification stability of the DQ-25 primers is not affected by the background of the rubber tree DNA template (Figure S3), indicating their potential for in vivo detection of *E. quercicola* in rubber trees.

### 3.2. Linearity, Dynamic Range, and Stability of qPCR and ddPCR in Detecting E. quercicola

**Figure 2:**
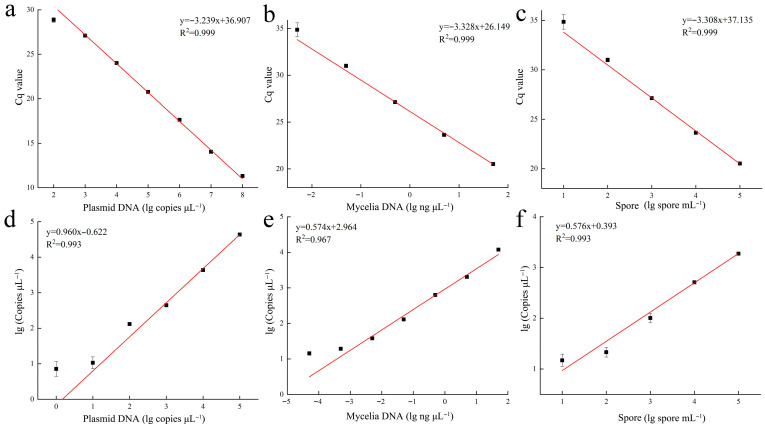
The standard curves of qPCR and ddPCR for plasmid DNA (**a**,**d**), *E. quercicola* mycelial DNA (**b**,**e**), and spore (**c**,**f**). The standard curve plots, slopes, Y-intercepts, and R^2^ values are shown. The X-axis values represent the 10-fold serial dilutions of DNA and spore.

**Figure 3:**
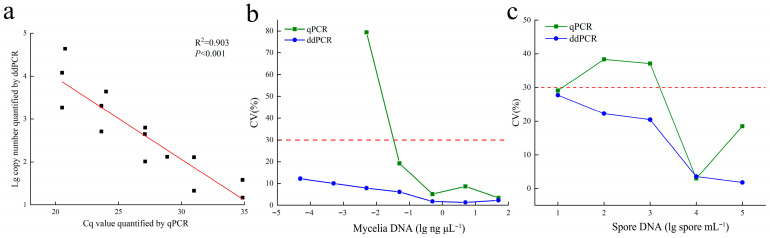
The stability of qPCR and ddPCR for *E. quercicola* mycelial DNA, and spore DNA. (**a**) Correlation between copy numbers measured by ddPCR and Cq values obtained from qPCR. (**b**) Coefficient of Variation (CV) for qPCR and ddPCR values for mycelial DNA across three quantitative repetitions. (**c**) CV for qPCR and ddPCR values for spore DNA across three quantitative repetitions.

This study evaluated the effectiveness of qPCR and ddPCR in detecting three types of samples: plasmid DNA containing the target fragment, *E. quercicola* mycelial DNA, and spore DNA. Linear fits between the concentration of DNA in the test samples and the detected DNA concentration yielded correlation coefficients (R^2^) consistently above 0.96 (Figure 2), demonstrating the high linearity of the established qPCR and ddPCR methods. Regarding dynamic range, qPCR detected plasmid DNA concentrations ranging from 10^2^ to 10^8^ copies/μL, while ddPCR detected concentrations between 1 to 10^5^ copies/μL. For mycelial DNA, qPCR detected concentrations from 10^−3^ to 10 ng/μL, whereas ddPCR detected from 10^−5^ to 10 ng/μL. In detecting spore DNA, both methods were capable of detecting concentrations from 10 to 10^5^ spores/mL. Additionally, the values detected by both methods show a significant correlation (*p* < 0.001; Figure 3a), indicating that the quantitative results from both methods are consistent and accurate. In terms of stability, the coefficient of variation for qPCR significantly increased in mycelial and spore samples with low DNA concentrations, exceeding 30%, whereas the coefficient of variation for ddPCR remained consistently lower than that for qPCR across various testing conditions, indicating greater stability (Figure 3b,c).

### 3.3. The Tolerance of qPCR and ddPCR to Isopropanol and Rubber Tree Sap

**Figure 4:**
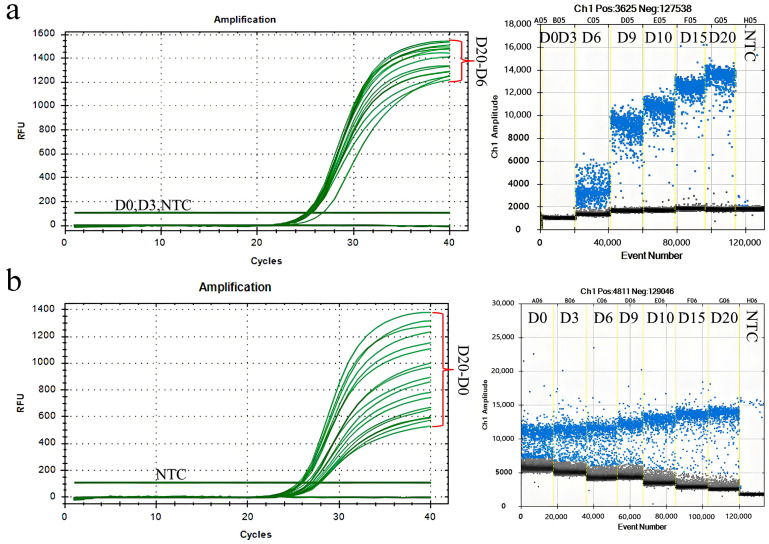
Tolerance of qPCR and ddPCR to isopropanol (**a**) and rubber tree sap (**b**). The letter “D” represents dilution treatments, with numbers indicating the dilution factors; “NTC” stands for the double-distilled water control.

To evaluate the tolerance of qPCR and ddPCR to amplification inhibitors in practical applications, this study utilized isopropanol and rubber tree sap as inhibitors. In the isopropanol tolerance tests, various concentrations of isopropanol were used to replace ddH_2_O in the reaction system (Figure 4a). The results showed that undiluted and 3-fold diluted isopropanol directly inhibited qPCR amplification. However, dilutions from 6-fold to 20-fold did not significantly alter the Cq values, but primarily affected the intensity of the fluorescence signals. Specifically, the smaller the dilution factor, the weaker the fluorescence signal produced during amplification (Figure 3a). In the tolerance tests with rubber tree sap, as the dilution factor decreased, the Cq values tended to increase significantly, and the intensity of the fluorescence signals decreased (Figure 4b). For ddPCR, both undiluted and 3-fold diluted isopropanol directly inhibited the amplification, while higher dilutions showed no significant impact on ddPCR effectiveness (Figure 4a). Additionally, the dispersion between positive and negative droplets increased as the dilution factor of isopropanol increased. Similarly, using rubber tree sap as an inhibitor did not alter ddPCR values, but increasing dilution resulted in greater dispersion between the two types of droplets (Figure 4b).

### 3.4. Quantifying E. quercicola Spores in Spore Trap Samples Using qPCR and ddPCR

**Figure 5:**
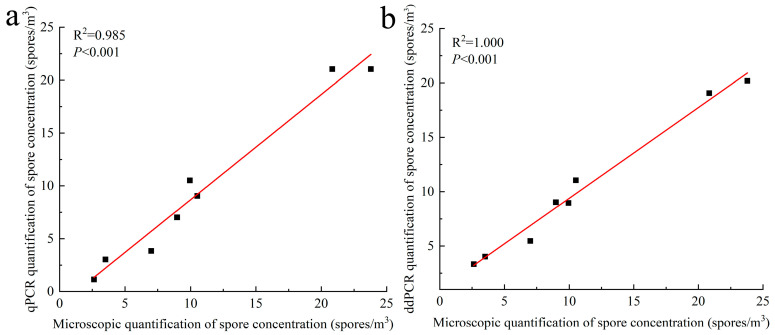
Correlation analysis between the quantification results of *E. quercicola* spores in rubber tree plantations using qPCR (**a**) and ddPCR (**b**), and microscopic observations. Quantification of *E. quercicola* spores collected in spore traps within rubber tree plantations was performed using qPCR and ddPCR. The results were analyzed for correlation with data obtained from microscopic observations. The data points represent the spore counts.

During the epidemic period of rubber tree powdery mildew, we collected eight spore trap samples in Yunnan and Hainan provinces. These samples allowed for the successful observation of *E. quercicola* conidiophores and facilitated the statistical analysis of spore density through microscopy. DNA was extracted from the conidiophores using a standard extraction method and subsequently quantified using qPCR and ddPCR. Additionally, we correlated the results from these two detection methods with the microscopic data (Figure 5). The spore concentrations measured using both platforms demonstrated excellent linearity (*p* < 0.001), yielding correlation coefficients of 0.985 for qPCR and 1.000 for ddPCR. These results indicate strong linear correlations and confirm the consistency between qPCR and ddPCR results and microscopic observations.

### 3.5. Early Detection of Powdery Mildew in Rubber Tree Saplings Using qPCR and ddPCR

**Figure 6:**
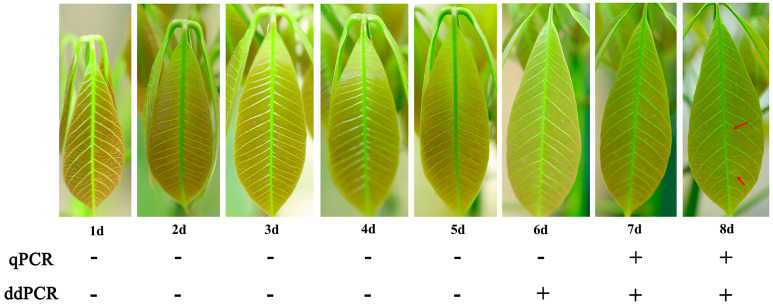
Detection of the natural disease progression in rubber saplings using qPCR and ddPCR. Rubber tree saplings were exposed to an environment with a spore concentration of approximately 10^3^ spores/m^3^ for natural inoculation. The seedlings were monitored daily for symptom development, and pathogen presence was confirmed on three selected leaves. Red arrows highlight visible fungal spots. The “+” symbol indicates detection of the fungus, and the “-” symbol indicates no fungus detected.

Rubber tree saplings were placed in a powdery mildew culture chamber with an approximate spore concentration of 10^3^ spores/m^3^ to allow for natural inoculation. By the eighth day after placement, sparse fungal colony spots were observed on the leaves (Figure 6). At the same time, the fungal load in the leaves was assessed using qPCR and ddPCR. The results showed that qPCR detected the DNA of *E. quercicola* one day before symptoms appeared, at a detection concentration of 25.1 copies/μL. ddPCR demonstrated higher sensitivity, detecting the DNA two days before the appearance of symptoms, at a detection concentration of 3.5 copies/μL.

### 3.6. Optimization and Validation of PMA Treatment for Detecting Fungal Viability Using qPCR and ddPCR

**Figure 7:**
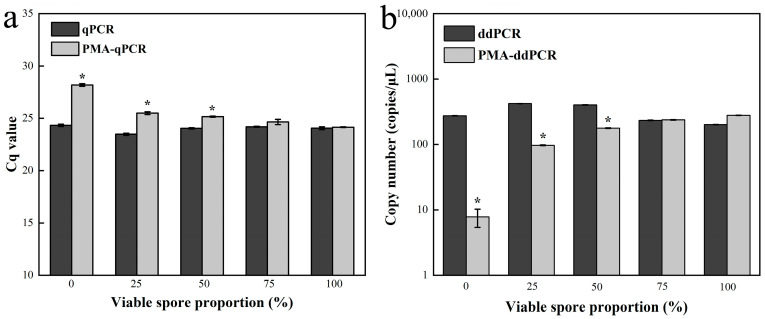
Detection of viable *E. quercicola* spores using PMA-qPCR and PMA-ddPCR. Live *E. quercicola* spores were mixed with dead spore samples in five different proportions, ranging from 0% to 100%. Each mixture was analyzed using both qPCR and ddPCR, with and without PMA. (**a**) Cq values obtained by qPCR and PMA-qPCR; (**b**) copy numbers (copies/µL) obtained by ddPCR and PMA-ddPCR. * Significant differences in spore numbers detected by the two methods, with or without PMA, were observed (*t*-test, *p* < 0.05).

We systematically optimized the conditions for fungal inactivation, PMA concentration, and duration of light exposure (Figure S4). The finalized protocol includes the following main steps: initially, mycelia or spores are treated with hot water at 95 °C for 10 min; subsequently, they are incubated with 40 µmol/L PMA for 15 min; finally, they are exposed to a 400 W halogen lamp for another 15 min. Viable and dead spores of *E. quercicola* were mixed in five different ratios, ranging from 0 to 100%, and then subjected to PMA treatment, followed by detection using qPCR and ddPCR. The results revealed significant differences (*p* < 0.05) in the detection outcomes between PMA-treated and untreated samples with identical concentration templates, particularly in samples with 0–50% viable spore ratios (Figure 7). These differences decreased as the proportion of live spores increased, demonstrating that PMA treatment, combined with qPCR and ddPCR, can effectively distinguish between viable and dead spores. Moreover, the detection sensitivity of PMA-ddPCR proved higher than that of PMA-qPCR, resulting in more pronounced differentiation effects.

### 3.7. Detecting Viable Fungi in Aged Lesions of Rubber Tree Powdery Mildew Using PMA-qPCR and PMA-ddPCR

**Figure 8:**
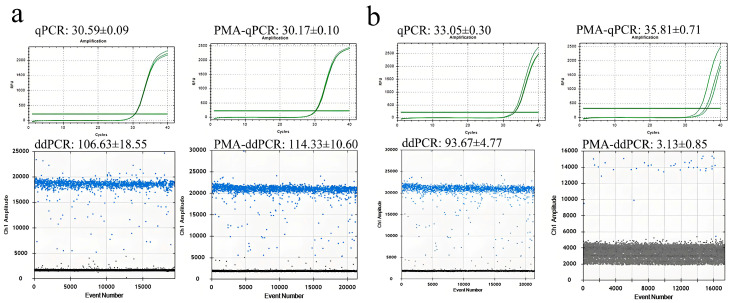
Quantification of viable fungi in fresh (**a**) and aged (**b**) powdery mildew lesions on rubber trees using PMA-qPCR and PMA-ddPCR. Fresh and aged powdery mildew spots on rubber tree leaves, which were artificially inoculated indoors, were collected. The lesioned leaves were analyzed using qPCR and ddPCR, with and without PMA. Copy numbers measured by ddPCR and Cq values obtained from qPCR are displayed.

In sections of rubber tree plantations affected by powdery mildew, numerous aged lesions are present on the leaves, which are devoid of any powdery substance. Despite their appearance, these lesions might harbor a certain amount of viable fungi, serving as potential sources for reinfection. This study employed PMA-qPCR and PMA-ddPCR to detect and assess viable fungi in fresh and aged powdery mildew lesions on rubber trees (Figure 8). The results showed that qPCR, ddPCR, PMA-qPCR, and PMA-ddPCR consistently detected rubber tree powdery mildew in fresh lesion samples, with similar quantitative values. In aged lesions, although qPCR and ddPCR continued to detect significant amounts of powdery mildew, the detected concentrations significantly decreased after PMA treatment. This indicates that PMA effectively inhibits the PCR amplification of DNA from dead fungi within the lesions. Therefore, PMA-qPCR and PMA-ddPCR prove to be effective in detecting viable fungi in the residues of rubber tree powdery mildew.

### 3.8. Assessing Fungicide Effectiveness Against Rubber Tree Powdery Mildew Using PMA-qPCR and PMA-ddPCR

**Figure 9:**
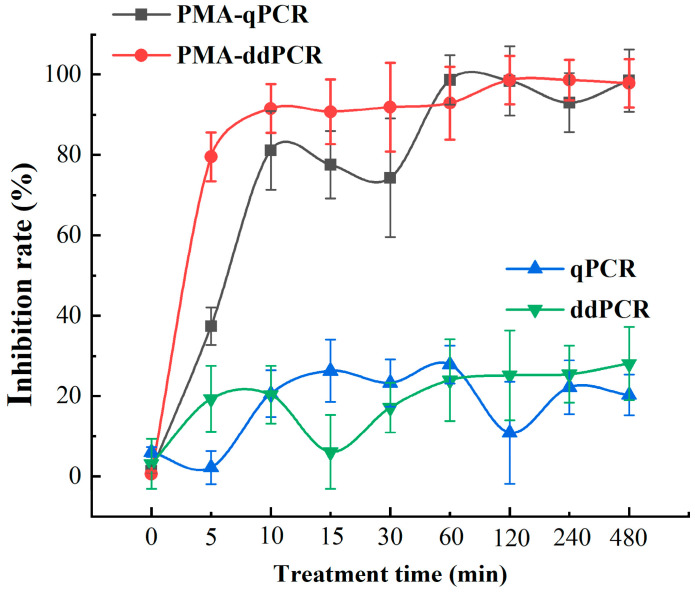
Detection of the inhibition effects of triadimefon on *E. quercicola* using PMA-qPCR and PMA-ddPCR. After the plants exhibited symptoms of powdery mildew the diseased leaves were sprayed with a 15% triadimefon wettable powder. Samples from the disease spots were collected at specific intervals during ten treatment periods. Samples not treated with triadimefon served as controls for the PMA-qPCR and PMA-ddPCR analyses.

This study investigated the capabilities of PMA-qPCR and PMA-ddPCR techniques in assessing the effectiveness of fungicidal treatments against rubber tree powdery mildew. Fungal detection on leaves treated with triadimefon was conducted. Ten minutes after the application of the triadimefon spray, the content of *E. quercicola* mycelia detected by both PMA-qPCR and PMA-ddPCR showed a significant decrease, indicating an inhibition rate of live fungi of at least 80%. In contrast, the inhibition rates observed using traditional qPCR and ddPCR were only around 20% and did not improve with prolonged treatment time. These findings indicate that the PMA-qPCR and PMA-ddPCR detection systems can rapidly quantify the number of viable *E. quercicola* mycelia following triadimefon treatment (Figure 9), thereby effectively evaluating the fungicidal control of rubber tree powdery mildew.

## 4. Discussion

Since the early 1950s, China’s rubber-growing regions have employed disease monitoring and spore trapping methods to guide the prediction and management of powdery mildew, achieving significant social and economic benefits. Disease surveys are labor- and resource-intensive, and while spore trapping, which involves microscopic examination of airborne fungal spores, is widely used, it is time-consuming and susceptible to interference from spore shrinkage and overlap, reducing detection accuracy [17]. PCR technology, which allows rapid in vitro amplification of pathogen-specific gene fragments, has provided a novel approach for detecting pathogens such as fungi, bacteria, and viruses. Currently, nested PCR and loop-mediated isothermal amplification have been applied for the detection of *E. quercicola* in rubber trees [8]. Although these methods improve detection speed, they still face limitations such as high false-positive rates, low sensitivity, and dependence on primer efficiency and standard curves, which prevent truly absolute quantification.

In pathogen molecular detection, qPCR technology offers broader development prospects and more widespread applications than other molecular detection methods. qPCR can perform quantitative analysis of pathogen DNA, which is a crucial technique in the study of plant disease epidemiology [18]. However, absolute quantification using qPCR has limitations: it relies on standard samples or curves to translate Cq values into the initial DNA template copy number, making the results relative. Additionally, factors such as primer amplification efficiency, the height of fluorescence thresholds, and PCR inhibitors can cause significant fluctuations in Cq values [19,20]. This variability makes it unfeasible to base results solely on the magnitude of Cq values, often necessitating subjective assessments by detection personnel and thus failing to achieve true “absolute quantification” [21]. As a third-generation PCR technology, ddPCR offers higher sensitivity, does not rely on standard curves, provides better repeatability, and demonstrates more stable performance in detecting low-concentration samples [22], allowing for the precise monitoring of minor changes in target sample concentrations [12]. The unique advantages of ddPCR have attracted increasing attention from scholars and have been extensively researched in fields such as tumor and cancer studies, viral and microbial detection analysis, and food safety monitoring [23,24,25]. In recent years, ddPCR has gradually been applied in agricultural production, emerging as a new tool for plant pathogen diagnosis and plant protection [13,26,27].

The specificity, linearity, dynamic range, stability and inhibitor tolerance of the qPCR and ddPCR detection systems are key factors that determine their detection capabilities. By designing primers specific to the *E. quercicola* sequence from rubber trees [28], we effectively addressed the issue of insufficient specificity when detecting this pathogen using both methods (Figure 1). In this study, we compared the dynamic range of qPCR and ddPCR using DNA templates of various concentrations from plasmid, mycelium, and spore samples. The quantitative results showed that both methods are consistent and accurate for detecting *E. quercicola* mycelia and spores (Figure 2). Owing to the limitation of positive droplet saturation, ddPCR cannot detect high concentrations of DNA; in contrast, qPCR can handle higher DNA concentrations. However, ddPCR has shown significantly higher sensitivity compared to qPCR. In this study, ddPCR’s detection sensitivity is typically 10–100 times higher than that of qPCR, which aligns with findings in detection systems for *Candidatus* Liberibacter asiaticus, *Acidovorax citrulli*, and *Xanthomonas citri* [29,30,31]. Regarding stability, a reduction in template concentration affects qPCR’s amplification efficiency, leading to decreased reproducibility; in contrast, ddPCR disperses one or a few target molecules into nanoliter-scale droplets using a droplet generator for independent amplification reactions, thus maintaining amplification efficiency and providing greater stability [32]. Consequently, ddPCR exhibits superior stability in detecting low-concentration templates compared to qPCR (Figure 3). During the extraction of pathogen DNA from plants, isopropanol precipitation is typically used, but plant pigments in the tissue fluids, which are difficult to remove, act as inhibitors that affect PCR amplification and consequently reduce sensitivity [33,34]. In this study, isopropanol and rubber tree sap were selected as amplification inhibitors in this study to provide a comprehensive assessment of the detection methods’ robustness. The results indicated that ddPCR exhibits greater tolerance than qPCR, effectively resisting interference from certain concentrations of inhibitors (Figure 4). These results suggest that ddPCR is more suitable for detecting *E. quercicola* in rubber tree leaves or in more complex environmental samples.

In forest sections susceptible to rubber tree powdery mildew, this study utilized spore traps to collect *E. quercicola* spores and employed qPCR and ddPCR for quantitative analysis, thereby validating the effectiveness of these detection methods. The experimental results show that the detection values from these molecular methods correlate well with spore density data observed under a microscope (Figure 5), demonstrating their rapidity and reliability for monitoring *E. quercicola* spores in rubber plantations. This integration of spore trapping and molecular detection significantly reduces biases from subjective human observation and provides robust detection capabilities at low spore concentrations, essential for implementing early control measures to prevent disease spread [35,36]. The ddPCR method, with its high sensitivity and stability in detecting low-concentration DNA, is especially suited for early-stage disease monitoring and precise quantification. For example, at the disease’s onset, the pathogen content in rubber tree leaves may be very low, yet ddPCR can detect these pathogens earlier (Figure 6), offering a scientific basis for the timely implementation of control measures. Moreover, PMA technology has been introduced to overcome the limitations of qPCR and ddPCR in distinguishing between live and dead cells [37]. After PMA treatment, qPCR and ddPCR can accurately quantify the number of viable spores (Figure 7), enabling more precise quantification of infectious spores in spore traps. Additionally, this combined technique can clearly differentiate between live and dead mycelia on aged, necrotic leaves (Figure 8), aiding in the identification of potential sources of reinfection. Furthermore, integrating PMA treatment with qPCR or ddPCR enhances the accuracy of field fungicide effectiveness assessments (Figure 9), aiding researchers and agricultural technicians in measuring changes in live cell counts before and after treatment, thus supporting the scientific application of pesticides in rubber plantations.

The integration of qPCR, ddPCR, and PMA treatments offers a novel and effective solution for detecting low-abundance *E. quercicola* in rubber tree powdery mildew, effectively addressing the limitations of traditional PCR methods. However, the high costs and complex operations associated with these advanced molecular techniques, which require specialized equipment and technical expertise, may restrict their adoption in resource-constrained settings [32]. To address these barriers, future research should aim to develop more affordable and user-friendly diagnostic tools. Enhancing the accessibility of these technologies through simplified equipment and providing extensive online training and support can broaden their application across various regions.

## 5. Conclusions

This study has successfully developed and validated a molecular detection platform integrating qPCR, ddPCR, and PMA treatments, significantly enhancing the detection and monitoring of *E. quercicola* in rubber trees. By combining advanced molecular techniques with traditional methods, we have increased the precision of pathogen detection and quantification, essential for effective disease management in rubber plantations. The optimized primers for qPCR and ddPCR exhibit high specificity and sensitivity, distinctly identifying *E. quercicola* from other pathogens. The platform demonstrated a robust detection range, quantifying *E. quercicola* accurately at concentrations as low as 10 spores/mL spore DNA and 10^−5^ ng/μL mycelial DNA. This accuracy facilitates early intervention and reduces dependence on fungicides, thereby minimizing both economic and environmental impacts. Furthermore, PMA treatments refine the detection process by differentiating between viable and non-viable fungal cells, a critical aspect for understanding disease epidemiology and assessing fungicide effectiveness. These advancements underscore the potential of molecular detection technologies in managing powdery mildew and highlight the need for continuous improvements to cope with complex field conditions, promising enhanced disease control and significant agricultural benefits.

## Data Availability

The original contributions presented in this study are included in the article/Supplementary Materials. Further inquiries can be directed to the corresponding authors.

## Supplementary Materials

The following supporting information can be downloaded at: https://www.mdpi.com/article/10.3390/jof12030185/s1, Figure S1: Fresh lesion (**a**) and aged lesion (**b**) of powdery mildew on rubber tree. Figure S2: The results of PCR (**a**) and ddPCR (**b**) annealing temperature screening. Figure S3: qPCR amplification standard curves with ddH_2_O as the background (**a**) and rubber tree DNA as the background (**b**). Figure S4: The effects of different inactivation methods (**a**), PMA treatment concentrations (**b**), and light exposure durations (**c**) on the efficacy of PMA treatment. * Significant differences in Cq values detected by three inactivation methods were observed (*t*-test, *p* < 0.05). Values followed by the same letter are not significantly (LSD, *p* < 0.05) different from one another. Table S1: Information of fungal strains used in this study. Table S2: Specific primers used in this study. Table S3: The results of qPCR annealing temperature screening.

## Abbreviations

qPCR	Real-time quantitative PCR
ddPCR	droplet digital PCR
PMA	propidium monoazide
gDNA	Genomic DNA
NTC	non-template control
CV	coefficient of variation
LSD	The Least significant difference
PMA-qPCR	Propidium Monoazide-quantitative Polymerase Chain Reaction
PMA-ddPCR	Propidium Monoazide-droplet digital Polymerase Chain Reaction
