# Supplementary material for: Enhanced Early Detection and Precision Monitoring of Rubber Tree Powdery Mildew Pathogen Erysiphe quercicola Using Quantitative PCR and Droplet Digital PCR
Source: J Fungi (Basel). 2026 Mar 5;12(3):185. doi: 10.3390/jof12030185 (PMC13028011; doi:10.3390/jof12030185)
Supplement: Supplementary file 1 [file jof-12-00185-s001.zip › jof-4130226-supplementary.pdf]

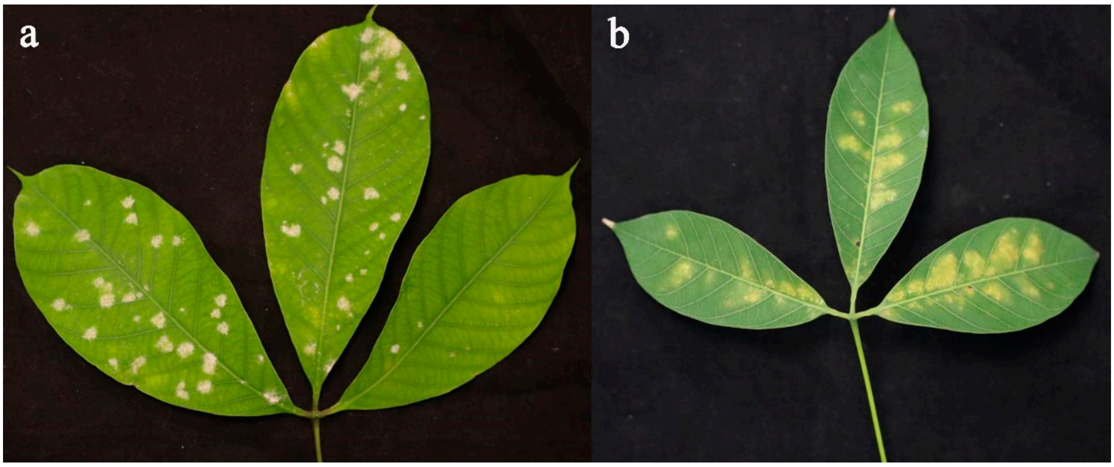

Figure S1. Fresh lesion (a) and aged lesion(b) of powdery mildew on rubber tree.

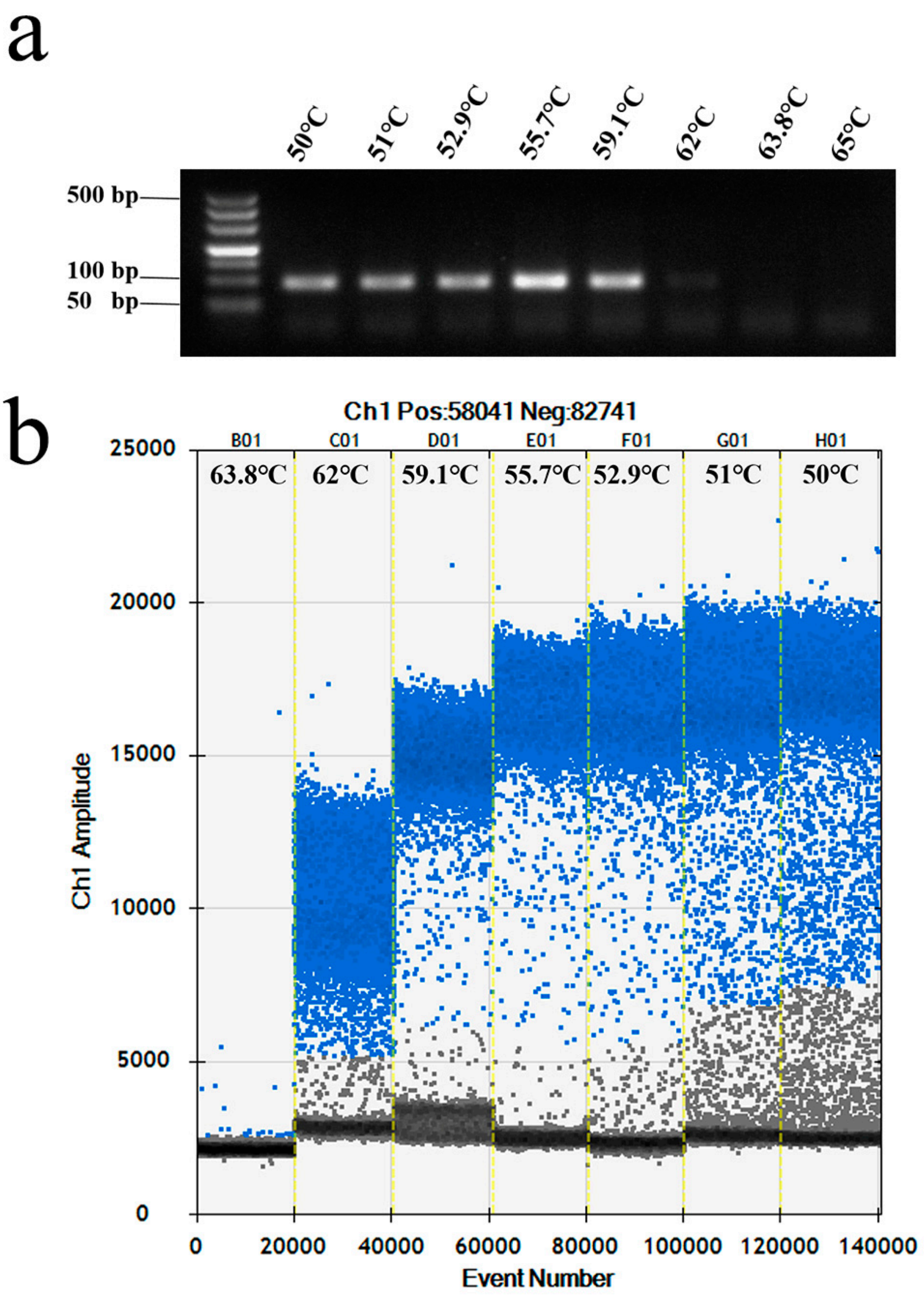

Figure S2. The results of PCR(a) and ddPCR(b) annealing temperature screening.

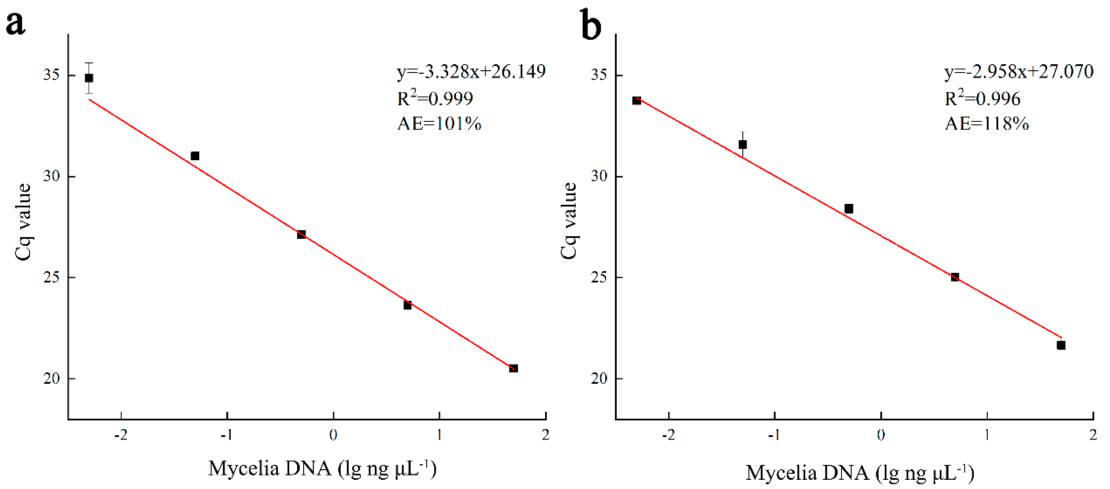

Figure S3. qPCR amplification standard curves with ddH_2O as the background (a) and rubber tree DNA as the background (b).

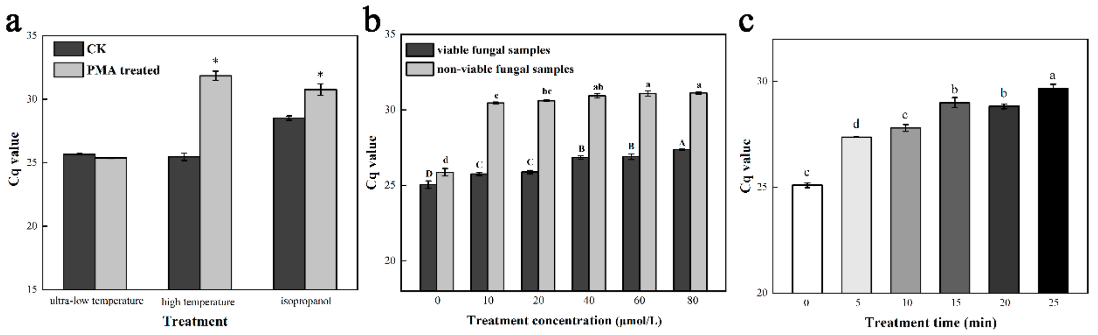

Figure S4. The effects of different inactivation methods (a), PMA treatment concentrations (b), and light exposure durations (c) on the efficacy of PMA treatment. *Significant differences in Cq values detected by three inactivation methods were observed (t-test, $P < 0.05$). Values followed by the same letter are not significantly (LSD, $P < 0.05$) different from one another.

Table S1. Information of fungal strains used in this study.

No.	Species	Host	Disease
1	*Erysiphe quercicola*	rubber tree	powdery mildew
2	*Erysiphe polygoni*	cowpea	powdery mildew
3	*Podosphaera xanthii*	bitter gourd	powdery mildew
4	*Corynespora cassiicola*	rubber tree	leaf fall
5	*Colletotrichum siamense*	rubber tree	anthracnose
6	*Phytophthora palmivora*	rubber tree	leaf fall

Table S2. Specific primers used in this study.

No.	Primer name	Sequence (5′-3′)	Product length (bp)
1	DQ-25F	ACCCCATCACGACTAAAATA	98
	DQ-25R	GGAAGTGGACCGACGAA	
2	DQ-12F	ATCAGCAGGACGACTGGGTG	91
	DQ-12R	TAGGTCTCGGGGTGGTATG	
3	DQ-13F	ACACCCCATCACGACTAAAAT	93
	DQ-13R	TGTAGGAAGTGGACCGACGAA	

Table S3. The results of qPCR annealing temperature screening.

Annealing temperature (℃)	Cq value	Fluorescence thresholds (RFU)
65	ND	-3.19
63.8	ND	2004
62	31.19	3633
59.1	23.66	4138
55.7	22.89	4605
52.9	22.71	4257
51	22.45	3845
50	22.54	3513
